# Formation of functional Tat translocases from heterologous components

**DOI:** 10.1186/1471-2180-6-64

**Published:** 2006-07-19

**Authors:** Matthew G Hicks, David Guymer, Grant Buchanan, David A Widdick, Isabelle Caldelari, Ben C Berks, Tracy Palmer

**Affiliations:** 1 Department of Molecular Microbiology, John Innes Centre, Norwich NR4 7UH, UK; 2 School of Biological Sciences, University of East Anglia, Norwich NR4 7TJ, UK; 3 Department of Biochemistry, University of Oxford, South Parks Road, Oxford OX1 3QU, UK

## Abstract

**Background:**

The Tat pathway transports folded proteins across the cytoplasmic membrane of bacteria and the thylakoid membrane of plants. In *Eschericha coli*, Tat transport requires the integral membrane proteins TatA, TatB and TatC. In this study we have tested the ability of *tat *genes from the eubacterial species *Pseudomonas syringae*, *Streptomyces coelicolor *and *Aquifex aeolicus*, to compensate for the absence of the cognate *E. coli tat *gene, and thus to form functional Tat translocases with *E. coli *Tat components.

**Results:**

All three subunits of the Tat system from the Gram positive organism *Streptomyces coelicolor *were able to form heterologous translocases with substantive Tat transport activity. However, only the TatA and TatB proteins of *Pseudomonas syringae *were able to functionally interact with the *E. coli *Tat system even though the two organisms are closely related. Of the Tat components from the phylogenetically distant hyperthermophillic bacterium *Aquifex aeolicus *only the TatA proteins showed any detectable level of heterologous functionality. The heterologously expressed TatA proteins of *S. coelicolor *and *A. aeolicus *were found exclusively in the membrane fraction.

**Conclusion:**

Our results show that of the three Tat proteins, TatA is most likely to show cross-species complementation. By contrast, TatB and TatC do not always show cross-complementation, probably because they must recognise heterologous signal peptides. Since heterologously-expressed *S. coelicolor *TatA protein was functional and found only in the membrane fraction, it suggests that soluble forms of *Streptomyces *TatA reported by others do not play a role in protein export.

## Background

There are two general pathways by which proteins are translocated across the cytoplasmic membranes of bacteria. The Sec pathway, which is ubiquitous, uses a threading mechanism to transport unfolded polypeptides across the membrane, driven by the energy of ATP hydrolysis and the transmembrane proton gradient [1]. By contrast, the Tat pathway, which is encoded in about half of bacterial genomes sequenced so far, exports only pre-folded proteins. Substrate proteins are targeted to the Tat machinery by N-terminal signal peptides that harbour an almost invariant pair of arginine residues, which are critical for transport by the Tat pathway [reviewed in [2,3]]. Tat transport is driven solely by the protonmotive force [4].

Much of our understanding of protein transport by the Tat pathway has come from dual studies of the bacterial Tat pathway and the homologous ΔpH/Tat pathway in plant thylakoids. The Tat system of *Escherichia coli *is comprised of the three major components, TatA, TatB and TatC, along with the minor component TatE which is a poorly expressed TatA orthologue [5-9]. Protein purification and cross-linking studies have identified two major types of Tat protein complexes in the membranes of *E. coli*, and analogous complexes have also been identified in thylakoid membranes. An equimolar complex of TatB and TatC, which contains multiple copies of each component, acts as the receptor for Tat substrates [10-12]. Site-specific cross-linking studies have implicated TatC as the component that recognizes the twin arginine motif of the substrate signal peptide [12]. The TatA protein forms a separate, highly heterogeneous complex, which varies in size because it contains different numbers of TatA protomers [13-16]. Examination of purified TatA complexes by negative stain electron microscopy reveal that it forms channel complexes with internal diameters large enough to accommodate folded substrate proteins [16]. Cross-linking studies suggest that TatA transiently associates with the substrate-bound TatBC complex during active protein translocation [12,17,18].

The Tat systems of some Gram positive bacteria, exemplified by *Bacillus subtilis*, and some Archaea show a slightly different organization in that they lack TatB and therefore have translocases that are comprised solely of TatA and TatC [19]. The structural arrangement of subunits in these minimal Tat systems is currently unknown. However, a number of reports have indicated that at least some of the TatA protein of *Haloferax volcanii*, *B. subtilis*, and of the TatA and TatB proteins of *Streptomyces lividans *exists in a soluble form in the cytoplasmic fractions of these organisms [20-22]. These findings are significant, because they imply that the Tat systems in these prokaryotes may operate by a somewhat different mechanism to the canonical Tat systems of *E. coli *and plant thylakoids.

Previous studies looking at heterologous interactions during Tat transport have generally focused on the ability of Tat systems to recognize and transport foreign Tat substrates. Thus *tat *genes from different bacterial sources have been expressed in an *E. coli *strain deleted for all Tat components [23], which inform on the capacity of foreign translocases to recognize and transport *E. coli *Tat substrate proteins. Conversely, foreign Tat substrates have also been expressed in *E. coli*, to test the capacity of the system to recognize non-native signal peptides and passenger proteins [e.g. [24-28]]. However, very few studies have looked at the ability of individual Tat subunits to substitute for the absence of the cognate *E. coli *Tat component. It was reported that *Helicobacter pylori tatA *could partially complement the Tat defect of an *E. coli *Δ*tatA*Δ*tatE *strain, but that *H. pylori tatB *could not substitute for *E. coli tatB *[8]. A very recent study suggested that the *P. syringae *pv *tomato *DC3000 *tatC *gene could also complement the *E. coli tatC *deletion strain [29].

In this work, we have systematically examined the ability of *tat *genes from three different bacterial species to compensate for the absence of the cognate *E. coli tat *gene. The organisms we selected for this study are *Aquifex aeolicus*, a thermophilic bacterium which forms the deepest branch in bacterial phylogeny, *Streptomyces coelicolor*, a Gram positive actinomycete and *Pseudomonas syringae *pv *maculicola *ES4326, which, like *E. coli*, is a gamma Proteobacterium. *E. coli *diverged from *A. aeolicus *approximately 4 billion years ago, from *S. coelicolor *approximately 3.2 billion years ago and from *P. syringae *approximately 1.3 billion years ago [30]. The percentage identities of the Tat proteins from these organisms with the paralogous *E. coli *Tat proteins is shown in Table 1. Our results indicate that TatA proteins from any of these organisms are able to, at least partially, restore Tat activity to a strain lacking *E. coli tatA *and *tatE*. The *P. syringae tatB *and *S. coelicolor tatB *and *tatC *genes were also able to complement the cognate *E. coli tat *deletion strains. Cell fractionation experiments demonstrate that the heterologously expressed TatA proteins of *S. coelicolor *and *A. aeolicus *are found exclusively in the membrane.

## Results and discussion

### Experimental design

Throughout these experiments we have used three different tests to assess functionality of the Tat system, each of which depends upon the transport of one or more native *E. coli *Tat substrates. *E. coli tat *mutants show a pleiotropic cell envelope defect due to an inability to export two Tat-dependent periplasmic amidases, AmiA and AmiC, which are involved in cell wall remodelling [31,32]. Strains with an inactive Tat system are unable to grow on solid media in the presence of 2% SDS and therefore the ability to grow in the presence of this detergent is a qualitative indication of Tat function [33]. Likewise, *E. coli *is able to grow anaerobically using trimethylamine-*N*-oxide (TMAO) as an electron acceptor due to the Tat-dependent export of two enzymes, TMAO reductase (TorA, which is a soluble periplasmic protein) and dimethylsulphoxide (DMSO) reductase (DmsABC, which is membrane-bound, with its active site facing the periplasm [34]). Therefore the ability of strains to grow anaerobically on minimal media with glycerol as a carbon source and TMAO as sole electron acceptor is also a qualitative indicator of Tat functionality. Finally we have also assayed the activity of TMAO reductase in periplasmic extracts as a more quantitative assessment of the level of Tat functionality.

We have observed previously that the stoichiometry of Tat subunit expression is critical for activity of the Tat translocase. In particular, high level expression of TatB relative to TatA and TatC has a severe inactivating effect on the *E. coli *Tat system [8]. Thus in the following experiments we have routinely used *pcnB *derivatives of each of the *tat *mutant strains, which drastically lowers the copy number of plasmids with the ColE1 replicon, typically to less than 5 copies per cell [35,36].

### Heterologous expression of TatA proteins

**Figure 1:**
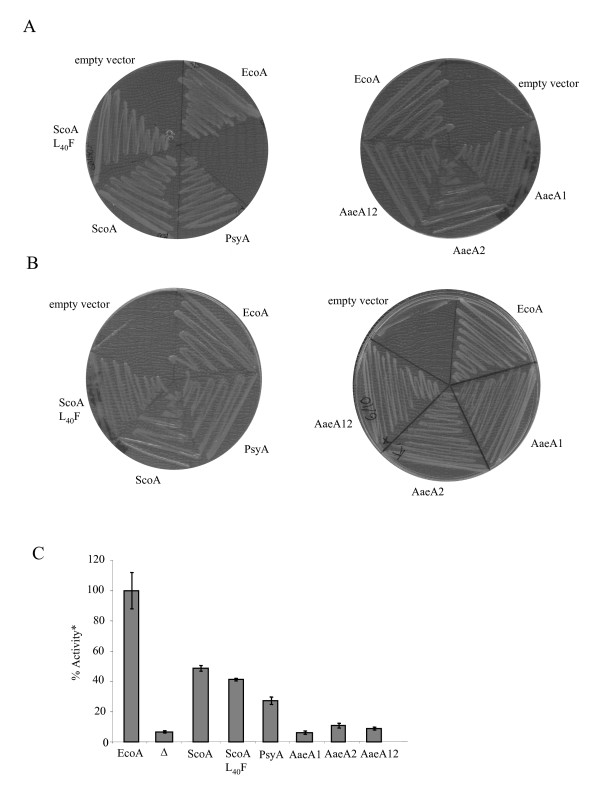
Complementation of the *E. coli *Δ*tatA*Δ*tatE*, *pcnB *strain, JARV16-P, with *tatA *genes from other bacteria. Strain JARV16-P was transformed with either: *E. coli tatA *encoded on pFAT415 (EcoA), *P. syringae tatA *on plasmid pUniprom-PA (PsyA), *S. coelicolor tatA *from plasmid pUniprom-SA (ScoA), or the L40F derivative (ScoA L_40_F), *A. aeolicus tatA1 *(AaeA1), *tatA2 *(Aae2) or *tatA12 *(Aae12) from pQEAQ1, pQEAQ2 and pQEAQ12, respectively, or pBluescript (empty vector; marked as Δ in panel C). A. Growth of strains on LB medium containing 2% SDS. B. Growth of strains anaerobically on minimal glycerol TMAO medium. C. TMAO reductase activities from periplasmic fractions. *100% activity is taken as that determined from the periplasmic fraction of JARV16-P carrying pFAT415 and corresponds to 1.3μmol benzyl viologen oxidised/min/mg protein. Error bars represent the standard error of the mean (n = 3).

As shown in Fig 1, expression of the *S. coelicolor *or *P. syringae *TatA proteins in the *E. coli tatA/tatE *mutant strain resulted in significant restoration of Tat system function. Thus the *tatA/tatE *strain expressing either of these constructs showed robust growth in the presence of SDS (Fig 1A), with TMAO as sole terminal electron acceptor (Fig 1B) and had significant TMAO reductase (TorA) activity in the periplasmic fraction (Fig 1C). *A. aeolicus *is unusual in that it has a tandem duplication of *tatA *genes, designated *tatA1 *and *tatA2*, which share 68% identity with each other over a 50 amino acid overlap. Expression of either or both of these *tatA *genes in the *E. coli tatA/tatE *strain was sufficient to support growth in the presence of SDS or with TMAO as sole electron acceptor (Fig 1A and 1B). However, the level of periplasmic TMAO reductase activity supported by these strains was not significantly above background (Fig 1C) indicating that the rate of TorA export was very low. None-the-less, these results suggest that TatA proteins from all of the three species tested are capable of some level of heterologous interaction with the *E. coli *TatBC proteins.

We have previously demonstrated that residue F39 of *E. coli *TatA is critical for TatA function and that mutation to anything other than tyrosine or tryptophan not only inactivates TatA function but also shows a dominant phenotype such that in co-expression studies it grossly affects the function of wild type TatA [37,38]. Surprisingly, *S. coelicolor *is one of a very few organisms that does not have phenylalanine at that position. Instead the amino acid at residue 40, which is the equivalent position in *S. coelicolor *TatA, is leucine. Although a leucine substitution of *E. coli *F39 is barely tolerated [38], the *S. coelicolor *TatA protein shows a significant level of functionality in *E. coli*. We tested whether the activity of *S. coelicolor *TatA could be enhanced by mutagenesis of L40 to phenylalanine. However, as shown in Fig 1C, this substitution did not significantly alter the functionality of *S. coelicolor *TatA in the *E. coli *system. We conclude that other residues in *S. coelicolor *TatA compensate for the presence of a leucine at this position.

### Heterologous expression of TatB

**Figure 2:**
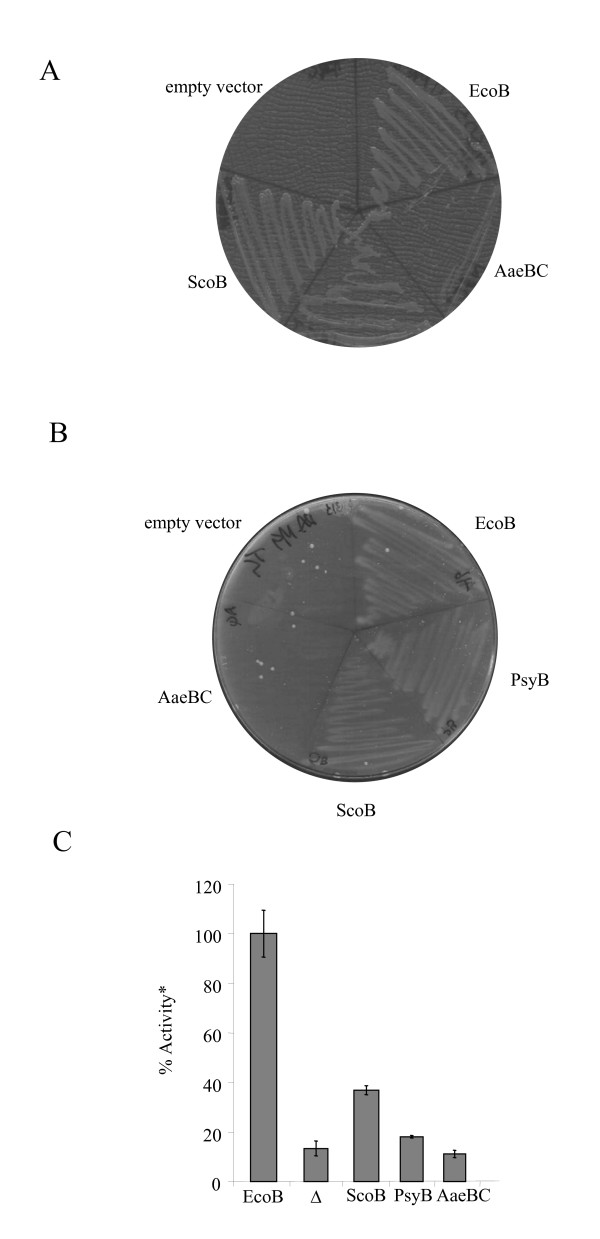
Complementation of the *E. coli *Δ*tatB, pcnB *strain, BØD-P, with *tatB *genes from other bacteria. Strain BØD-P was transformed with either: *E. coli tatB *encoded on pFAT416 (EcoB), *P. syringae tatB *on plasmid pUniprom-PB (PsyB), *S. coelicolor tatB *from plasmid pUniprom-SB (ScoB), *A. aeolicus tatBC *from plasmid pQEAQBC (AaeBC) or pBluescript (empty vector; marked as Δ in panel C). A. Growth of strains on LB medium containing 2% SDS. B. Growth of strains anaerobically on minimal glycerol TMAO medium. C. TMAO reductase activities from periplasmic fractions. *100% activity is taken as that determined from the periplasmic fraction of BØD-P carrying pFAT416 and corresponds to 0.83μmol benzyl viologen oxidised/min/mg protein. Error bars represent the standard error of the mean (n = 3).

The TatB protein, where present, is an essential component of the Tat system. It forms an equimolar complex with TatC and site-specific cross-linking analysis has indicated that it contacts Tat signal peptides close to the twin arginine motif, but also within the hydrophobic core [10,12]. In addition TatB has also been shown to co-purify with TatA, with which it shares some sequence homology [39]. It has been suggested to function as the adaptor between the TatBC complex and the TatA channel complex and therefore high level expression of TatB may disrupt Tat function by interfering with the co-assembly of the two individual complexes [8]. Since it interacts with each of the other Tat components and with Tat signal peptides, cross-complementation with TatB might be expected to be less efficient than with other Tat proteins. As shown in Fig 2A and 2B, the TatB proteins from either *S. coelicolor *or *P. syringae *permitted significant growth of the *E. coli tatB *strain, BØD-P, on selective media containing either SDS or TMAO. By contrast *A. aeolicus *TatB (co-expressed on a construct that also contains *tatC*) did not allow any significant growth of the *E. coli tatB *mutant on either medium. It should, however, be noted that we were not able to demonstrate expression of the *A. aeolicus *TatB protein in *E. coli *(data not shown) so it is possible that *A. aeolicus *TatB does not complement the *E. coli tatB *mutant because the protein is not produced. Fractionation of the *tatB *strain harbouring the different *tatB *genes and assay for periplasmic TMAO reductase activity, shown in Fig 2C, demonstrated that, as seen for TatA, the *S. coelicolor *TatB homologue gave the highest level of cross-species complementation.

It has been reported previously that the TatA and TatB proteins of *S. lividans*, an extremely close relative of *S. coelicolor *have partially overlapping activities [40]. We therefore tested the ability of the *S. coelicolor tatB *gene to complement the *E. coli tatA/E *mutant strain, and likewise the *S. coelicolor tatA *gene to complement the *E. coli tatB *mutant. In each case we saw no detectable complementation (results not shown).

### Heterologous expression of TatC

**Figure 3:**
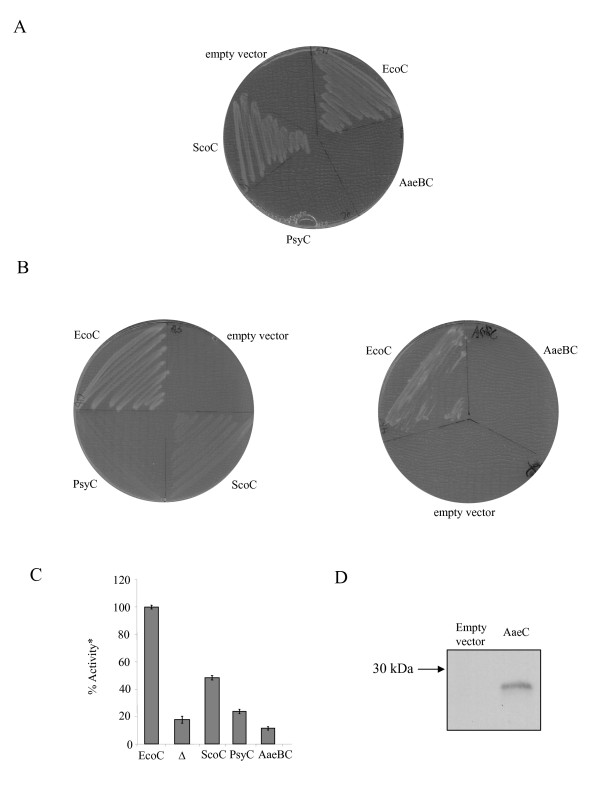
Complementation of the *E. coli *Δ*tatC, pcnB *strain, B1LK0-P, with *tatC *genes from other bacteria. Strain B1LK0-P was transformed with either: *E. coli tatC *encoded on pFAT417 (EcoC), *P. syringae tatC *on plasmid pUniprom-PC (PsyC), *S. coelicolor tatC *from plasmid pUniprom-SC (ScoC), *A. aeolicus tatBC *from plasmid pQEAQBC (AaeBC) or pBluescript (empty vector; marked as Δ in panel C). A. Growth of strains on LB medium containing 2% SDS. B. Growth of strains anaerobically on minimal glycerol TMAO medium. C. TMAO reductase activities from periplasmic fractions. *100% activity is taken as that determined from the periplasmic fraction of B1LK0-P carrying pFAT417 and corresponds to 0.79μmol benzyl viologen oxidised/min/mg protein. Error bars represent the standard error of the mean (n = 3). D. The *A. aeolicus *TatC protein is produced from clone pQEAQBC. Strain M15 [pREP4] harboring either pQE60 (empty vector) or pQEAQBC (AaeC) was cultured in LB medium until OD600 of 0.4 was reached, after which production of the TatChis protein was induced by addition of 1 mM isopropyl-β-D-galactopyranoside (IPTG) for a further 2 hours. Membrane fractions were prepared, proteins (50μg of total membrane protein), separated by SDS-PAGE, blotted onto nitrocellulose and developed using anti-penta-His antiserum.

The TatC protein is the largest and most conserved Tat component. It has been implicated as a specificity determinant for Tat-dependent secretion, most likely through recognition of Tat signal peptides [19,41]. Moreover, it has also been shown to cross-link with Tat signals when the site-specific cross-linker was incorporated adjacent to the twin arginines, suggesting that it probably recognizes the twin arginine motif of the signal peptide [12]. As shown in Fig 3A, B and 3C, only the TatC protein of *S. coelicolor *restored any detectable level of Tat activity to the *E. coli tatC, pcnB *mutant strain. The *tatC *genes of *P. syringae *and *A. aeolicus *(the latter co-expressed with *tatB*) completely failed to complement the *tatC *strain, even for growth on SDS-containing medium, which is the most sensitive test for native Tat substrate export [42]. As shown in Fig 3D, despite its lack of functionality when expressed in the *E. coli tatC *strain, the *A. aeolicus *his-tagged TatC protein encoded on our construct was clearly produced in *E. coli *and moreover was localized to the membrane fraction. We were not able to demonstrate whether the *P. syringae *TatC protein was expressed in these experiments because we lack a native antibody to the protein. However in the section below we demonstrate clearly that it is expressed from an analogous clone that also encodes *P. syringae tatA *and *tatB*. Taken together our results suggest that *S. coelicolor *Tat subunits show the greatest level of cross-complementation, even though this organism is more distantly related to *E. coli *than is *P. syringae*.

### Heterologous expression of tatABC genes in a strain of *E. coli *devoid of all Tat components

**Figure 4:**
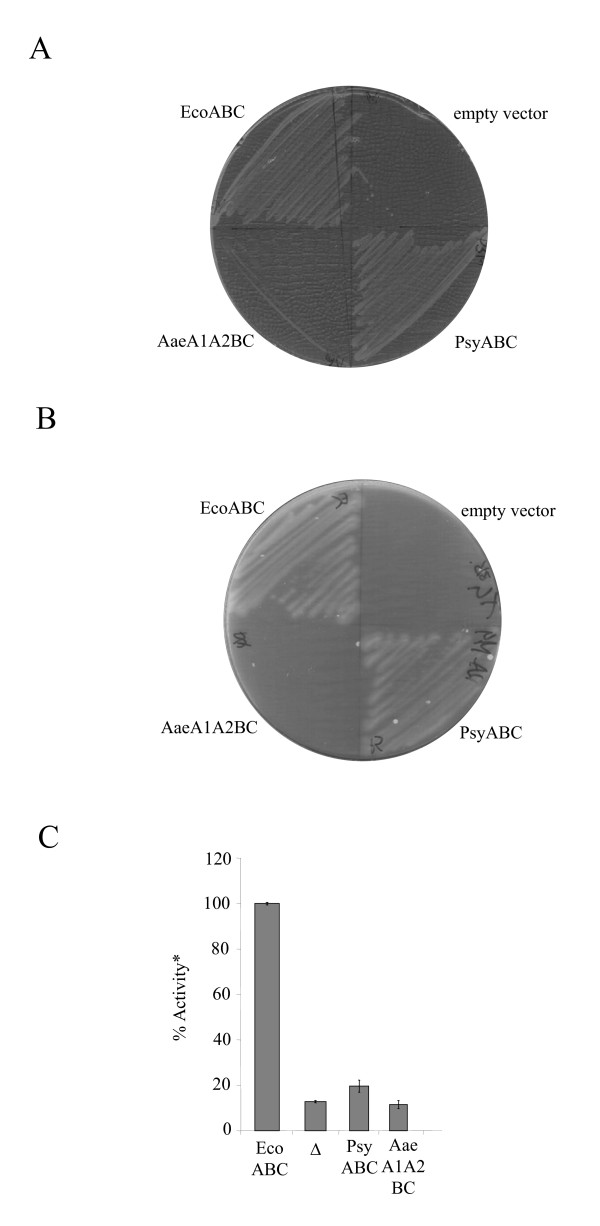
Complementation of the *E. coli *Δ*tatABCD*Δ*tatE, pcnB *strain, DADE-P, with *tatABC *genes from other bacteria. Strain DADE-P was transformed with either: *E. coli tatABC *encoded on pUnitat2 (EcoABC), *P. syringae tatABC *on plasmid pUniprom-PABC (PsyABC), *A. aeolicus tatA1A2BC *from plasmid pFATAQ3 (AaeA1A2BC) or pBluescript (empty vector; marked as Δ in panel C). A. Growth of strains on LB medium containing 2% SDS. B. Growth of strains anaerobically on minimal glycerol TMAO medium. C. TMAO reductase activities from periplasmic fractions. *100% activity is taken as that determined from the periplasmic fraction of DADE-P carrying pUnitat2 and corresponds to 0.83μmol benzyl viologen oxidised/min/mg protein. Error bars represent the standard error of the mean (n = 3).

The experiments described above where individual Tat subunits are expressed in *E. coli *examine the ability of that particular subunit to interact with *E. coli *Tat components, and, in addition, to recognize *E. coli *Tat signal peptides and substrate proteins. Under these circumstances, it is apparent that whilst all of the TatA proteins displayed some level of heterologous function, the *A. aeolicus tatB *and *tatC *genes, and *P. syringae tatC*, failed to complement the cognate *E. coli tat *mutant strains. In order to determine whether this was because of an inability to form functional complexes with *E. coli *Tat components rather than an inability to interact with the test *E. coli *substrates, we examined whether transport activity was observed when the full set of heterologous Tat proteins were co-expressed. As shown in Fig 4, the *P. syringae tatABC *operon showed a low but detectable level of Tat function, indicating that the *P. syringae *TatC protein alone was not functional in the *E. coli tatC *strain most likely because it could not either interact with *E. coli *TatB and/or TatA, or because it was expressed at an inappropriate ratio with these proteins. An alternative explanation is that *P. syringae tatC *in vector pUniprom-PC fails to express, although we note that it clearly does express from the same vector in the presence of *P. syringae tatA *and *tatB*. Conversely, the *A. aeolicus tatA1*, *tatA2*, *tatB *and *tatC *genes when expressed together from an artificial operon could not complement the *E. coli *total *tat *deletion strain. With the proviso that all of the *A. aeolicus tatA*, *tatB *and *tatC *genes express in *E. coli*, this result suggests that the *A. aeolicus *Tat proteins are unable to recognize any of the AmiA, AmiC, TorA or DmsA signal peptides.

A summary of the results we obtained for all of the heterologous expression experiments is shown in Table 2.

### Heterologously expressed TatA proteins from *S. coelicolor *and *A. aeolicus *are found exclusively in the membrane fraction

**Figure 5:**
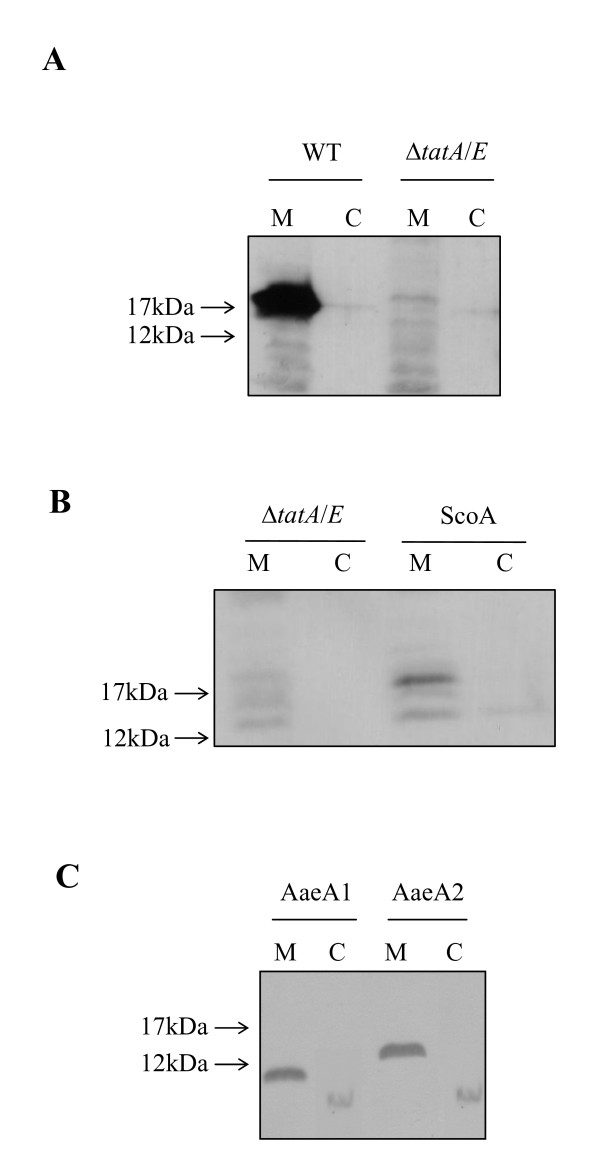
Heterologously expressed TatA proteins are located exclusively in the membrane. A. Strains MC4100 (*E. coli *parental strain; *tat*^+^; WT) and JARV16-P (as MC4100, Δ*tatA*/Δ*tatE*, *pcnB*; Δ*tatA*/*E*) were grown aerobically to late exponential phase in LB medium prior to harvesting and fractionation into membrane (M) and soluble (C) fractions. Samples were separated by SDS PAGE, electroblotted and probed with anti *E. coli *TatA antiserum. B. Strain JARV16-P (Δ*tatA*/*E*) or JARV16-P carrying pUniprom-SA encoding *S. coelicolor *TatA (ScoA) were cultured, fractionated and blotted as above, and probed with anti *S. coelicolor *TatA peptide antiserum. C. Strain M15 [pREP4] harboring either pQEAQ1 (AaeA1) or pQEAQ2 (AaeA2) encoding *A. aeolicus *TatA1 or TatA2 with C-terminal histags, respectively. Cells were cultured in LB medium until OD600 of 0.4 was reached, after which expression of the TatA protein was induced by addition of 1 mM isopropyl-β-D-galactopyranoside (IPTG) for a further 2 hours. Cells were fractionated and blotted as above, and probed with anti pentahis antiserum. For each panel, membrane and soluble material from an equivalent amount of cells was loaded.

One of the most striking reported differences between the Tat system of *E. coli *and of Gram positive (and archaeal) Tat systems is the presence of soluble forms of TatA in these latter organisms. Since we demonstrated above that the *S. coelicolor *and *A. aeolicus *TatA proteins show functionality in *E. coli*, it was reasonable to examine the subcellular location of these heterologously expressed TatA proteins. As shown in Fig 5A, all of the *E. coli *TatA protein was found in the membrane fraction. Strikingly, we also found all of the heterologously expressed *S. coelicolor *TatA and of the his-tagged *A. aeolicus *TatA1 and TatA2 proteins exclusively in the membrane fraction. Whilst we cannot rule out that any cytoplasmic forms of these heterologously-produced proteins might have been degraded, these observations indicate that at least when expressed in *E. coli *it is the membrane-bound protein that is functional for transport.

## Conclusion

In this report we have tested the ability of Tat proteins from three different eubacterial species to form complexes with *E. coli *Tat components. Surprisingly, we found that it was the individual Tat proteins of *S. coelicolor *that consistently gave the highest level of Tat activity with the cognate *E. coli tat *deletion strains. Indeed each of *S. coelicolor *TatA, TatB and TatC formed heterologous Tat systems that were able to support 40–50% of the wild type *E. coli *Tat activity. The *P. syringae *TatA protein was able to restore reasonable Tat function to the *E. coli tatA*/*E *mutant strain, but the *tatB *gene complemented very poorly and *P. syringae *TatC was apparently completely inactive.

For each of the *S. coelicolor *and *P. syringae *cross-complementation experiments, individual Tat subunits were expressed from identical constructs, with transcription being driven by the constitutive *E. coli tat *promoter. It is probable that the heterologously expressed *S. coelicolor tat *genes are translated less efficiently than the *P. syringae *genes because of codon bias resulting from the high G+C content of *S. coelicolor *DNA. Whilst we cannot completely rule out the possibility that there is a deleteriously high level of production of the individual *P. syringae *Tat proteins (but see below), it is none-the-less, striking that the *S. coelicolor *Tat proteins show a high level of cross-species activity. One possible explanation for this might be linked to the fact that the *S. coelicolor *has by far the largest predicted Tat secretome. Thus the *S. coelicolor *Tat system probably transports in excess of 150 Tat substrates, including some of the largest Tat substrates ever identified [43-45]. It therefore must recognize many different twin arginine signal peptides and as a result one might expect that the *S. coelicolor *Tat components are less stringent than the *P. syringae *system in terms of signal peptide recognition and substrate size.

It should be noted that TatC from a different pathovar of *P. syringae *to that tested here [but with which it shares 97% amino acid sequence identity; [46]] was reported to show at least some minimal Tat activity in the *E. coli tatC *strain for transport of the Tat substrates AmiA and/or AmiC [29]. However in those experiments, the expression of *P. syringae tatC *was from the strong p*tac *promoter on plasmid pTrc99A and was carried out in a *pcnB*^+ ^background where plasmid copy number would be considerably higher. Thus it is likely that the levels of TatC between the two experiments would be significantly different which may account for the discrepancy in the observed activities.

It is striking to note that wherever cross-species complementation has been tested, TatA proteins always seem to retain some level of function in the heterologous host [8,47]. This suggests that the constraints on TatA function are less severe than those for TatB or TatC and is consistent with the fact that most of the interactions of heterologously expressed TatA would be self-oligomerisation to assemble into channel-forming multimers. By contrast, the constraints on cross-complementation with heterologously expressed TatB or TatC proteins are likely to be much more stringent since this would require the formation of equimolar complexes with the appropriate *E. coli *partner subunit, recognition of non-native signal peptides and associated conformational changes to promote assembly of the active translocase. It is notable that the *A. aeolicus *TatA proteins, which would normally be operating at temperatures of 80°C, retain function when expressed in *E. coli *cells growing at 37°C. Thus either the TatA protein does not change conformation during the transport cycle or the protein retains sufficient flexibility for function even at temperatures considerably lower than physiological.

A number of groups have reported in the literature the presence of soluble forms of the TatA and TatB proteins from Gram positive bacteria and archaea [20-22]. Fractionation and Western blotting showed quite clearly here that all of the heterologously expressed TatA proteins from *S. coelicolor *and *A. aeolicus *are found only in the membrane fraction. It is not clear why extra-membraneous forms of TatA/TatB are present in some organisms, but clearly since the *S. coelicolor *TatA protein [which is 100% identical to the *S. lividans *TatA protein; [48]] supports such a high level of Tat activity in *E. coli *then it is difficult to imagine how the mechanism of Tat transport at least between *E. coli *and Streptomycetes cannot be anything other than highly similar.

## Methods

### Bacterial strains and growth conditions

The *E. coli *strains and plasmids employed in this study are shown in Table 3. During all genetic manipulations, *E. coli *strains were grown aerobically in Luria-Bertani (LB) medium [49]. Concentrations of antibiotics were as described previously [6]. The growth phenotypes of mutants with TMAO as sole respiratory electron acceptor were determined on M9 minimal medium agar plates [49] supplemented with 0.5% glycerol and 0.4% TMAO and incubated in a gas jar under a hydrogen/carbon dioxide atmosphere. The SDS-resistance phenotype of mutants was tested on LB agar plates containing 2% SDS [33]. For TMAO reductase assay, cells were cultured in modified Cohen and Rickenberg medium [50], supplemented with addition of 0.2% glucose and 0.4% TMAO.

### Plasmid construction

Plasmids pUniprom-PA, pUniprom-PB and pUniprom-PC carry the *P. syringae *pv *maculicola *ES4326 *tatA*, *tatB *and *tatC *genes, respectively, under the control of the *E. coli tat *promoter. They were cloned following amplification with primers GCGGCC**GGATCC**ATGGGTATTTTTGACTGG and GCGC**TCTAGA**TTAAACC TGGTCTTTGCGG to amplify *tatA*, GCGCGG**GGATCC**ATGTTCGGTATCAGC and GCGCGC**TCTAGA**TCATGGGGCTCGCGGTGGC to amplify *tatB*, and GCGC GC**GGATCC**ATGAGCGCTGATATCCCG and GCGCGC**TCTAGA**TCACGGTGT GGTGGCGGGCGG to amplify *tatC *(restriction sites shown in bold) and plasmid pKS-PSM*tatABC *[46] as template. Each product was digested with *Bam*HI and *Xba*I and cloned into pUniprom [51] that had been similarly digested. Plasmids pUniprom-SA, pUniprom-SB and pUniprom-SC carry the *S. coelicolor tatA*, *tatB *and *tatC *genes, respectively, under the control of the *E. coli tat *promoter. They were cloned following amplification with primers GCGCGC**GGATCC**ATGTTCGGAAGGCTCG GC and GCGCGC**TCTAGA**TCAGCGCTTGGTCGTGTC to amplify *tatA*, GCGCG C**GGATTC**GTGTTCAATGACATAGGC and GCGCGC**TCTAGA**TCAGGTGGCG TCCATGTC to amplify *tatB*, and GCGCGC**GGATCC**ATGCCGCTCGCGGAACA C and GCGCGC**TCTAGA**TCAGGTCACGTCGTCG to amplify *tatC *with *S. coelicolor *chromosomal DNA as template. Each product was digested with *Bam*HI and *Xba*I and cloned into pUniprom that had been similarly digested.

Plasmids pFATAQ1 contains the *tatA1 *and *tatA2 *genes of *A. aeolicus*, under control of the *lac *promoter. The *tatA1 *and *tatA2 *genes, whose reading frames overlap, were amplified with the following primers: GCGCGC**GAATTC**CCCTTAAATTATTCTC TAAGGAGGC and GCGCGC**GGATCC**GCTGAGTTAAGCCTCTACCTTTTCC, with *A. aeolicus *chromosomal DNA (a kind gift of R. Huber) as template. The product was digested with *Eco*RI and *Bam*HI and cloned into pBluescript that had been digested with the same enzymes. The *A. aeolicus tatB *gene is not annotated on the genome [52], however, a gene encoding a protein with 23% identity (over 102 amino acid overlap) to *E. coli *TatB is found immediately upstream of the *tatC *gene (see Table 1 for identities of Tat proteins studied in this work with the paralogous *E. coli *protein), and we herein refer to it as *tatB*. Plasmid pFATAQ2 contains the *tatB *and *tatC *genes of *A. aeolicus*, under control of the *lac *promoter. The genes, whose reading frames also overlap, were amplified with primers GCGC**GGATCC**GGAAA GCAATCCCTATTAACGGAAG and GCGC**TCTAGA**GCTTATGCCTTTTGAATT TCCTTC, digested with *Bam*HI and *Xba*I and cloned into pBluescript that had been pre-digested with the same enzymes. Plasmid pFATAQ3 contains an artificial operon encoding the *tatA1*, *-A2*, *-B *and *-C *genes of *A. aeolicus *and was generated by subcloning the *tatBC *coding region from pFATAQ2 by digestion with *Bam*HI and *Xba*I and ligation into pFATAQ1 that had been similarly digested. For overproduction of the *A. aeolicus *Tat proteins with C-terminal hexahistidine affinity tags, the encoding *tat *genes were re-amplified and cloned into the overexpression vector pQE70. Plasmid pQEAQ1 contains the gene encoding the TatA1 protein with a C-terminal histag and was amplified using primers GCGC**GCATGC**ACTTTCCTCTGC CGTGGC and GCGC**AGATCT**TTTCACCCTCCTTTTTAACTTCC, and pFATAQ1 as template. The product was digested with *Sph*I and *Bgl*II, and cloned into similarly digested pQE70. Plasmid pQEAQ2 is analogous to pQEAQ1 but encodes a tagged version of the TatA2 protein. It was cloned in a similar manner, using primers GCGC **ACATGT**TTTCCCGGCGGAATATCTATG and GCGC**AGATCT**AGCCTCTACC TTTTCCTTCTC to amplify the encoding gene. Plasmid pQEAQ12 carries both of the *A. aeolicus tatA *genes, with just TatA2 supplied with the C-terminal histag. The genes were amplified using primers GCGC**GCATGC**ACTTTCCTCTGCCGTGGC and GC GC**AGATCT**AGCCTCTACCTTTTCCTTCTC, the product digested with *Sph*I and *Bgl*II, and cloned into similarly digested pQE70.

All clones obtained from PCR amplified DNA were sequenced to ensure that no mutations (other than site-specific mutations) had been introduced.

### Protein methods

SDS-PAGE and immunoblotting were carried out as described [53,54] and immunoreactive bands were visualized with the ECL detection system (Amersham Biosciences). Peptide antibodies to the *S. coelicolor *TatA protein were raised in rabbits, using a peptide of amino acid sequence CTSSRPVTEPTDTTKR, by Davids biotechnology (Regensburg, Germany). Antibodies to *E. coli *TatA have been described previously [39], the anti-pentahis antibody was obtained from Qiagen. Subcellular fractions for TMAO reductase activity measurements were prepared from small (30 ml) cultures using the cold osmotic shock protocol [55]. TMAO:benzyl viologen oxidoreductase activity was measured as described previously [56]. Protein concentrations were estimated according to the method of Lowry *et al*. [57]. Preparation of membrane fractions was as described previously [6].

## Abbreviations

Sec -general secretory pathway, Tat – Twin arginine translocation pathway, TMAO -trimethylamine-*N*-oxide, TorA – TMAO reductase DMSO – dimethylsulphoxide, DmsABC – membrane-bound DMSO reductase.

## Authors' contributions

MGH, DG, GB, IC and DW undertook experiments, BCB and TP wrote the manuscript.

## Figures and Tables

**Table 1:** Percentage identities between *E. coli *Tat proteins and those from other eubacteria analysed in this study.

Protein	Length (amino acids)	Percentage identity to *E. coli *homologue
*E. coli *TatA	89	
*P. syringae *TatA	91	60% over 48 residue overlap
*S. coelicolor *TatA	95	36% over 40 residue overlap
*A. aeolicus *TatA1	59	41% over 46 residue overlap
*A. aeolicus *TatA2	77	38% over 55 residue overlap
*E. coli *TatB	171	
*P. syringae *TatB	153	40% over 71 residue overlap
*S. coelicolor *TatB	161	33% over 55 residue overlap
*A. aeolicus *TatB	117	23% over 102 residue overlap
*E. coli *TatC	258	
*P. syringae *TatC	266	62% over 245 residue overlap
*S. coelicolor *TatC	301	27% over 247 residue overlap
*A. aeolicus *TatC	240	41% over 234 residue overlap

**Table 2:** Summary of Tat activity observed after heterologous expression of *tat *genes from different bacteria in *E. coli tat *mutant strains.

*E. coli *strain	Plasmid-encoded gene(s)	Functionality of the Tat system^1^
JARV16-P(Δ*tatA*/Δ*tatE*)	*Eco tatA*	++
	*Psy tatA*	++
	*Sco tatA*	++
	*Sco tatA*(L_40_F)	++
	*Aae tatA1*	+
	*Aae tatA2*	+
BØD-P(Δ*tatB*)	*Eco tatB*	++
	*Psy tatB*	+
	*Sco tatB*	++
	*Aae tatB*	-
B1LK0-P(Δ*tatC*)	*Eco tatC*	++
	*Psy tatC*	-
	*Sco tatC*	++
	*Aae tatC*	-
DADE-P(Δ*tatABCD*/Δ*tatE*)	*Eco tatABC*	++
	*Psy tatABC*	+
	*Aae tatA1A2tatBC*	-

^1^++ indicates a high level of functionality (>30% of the wild type level of periplasmic TMAO reductase activity, + indicates a lower level of functionality (<30% of the wild type level of periplasmic TMAO reductase activity, but confers the ability to grow on solid media containing SDS or TMAO), – indicates a lack of activity (no growth on SDS or TMAO-containing plates).

**Table 3:** Strains and plasmids used in this study

Bacterial Strains	Genotype	Source
MC4100	F^-^Δ*lacU169 araD139 rpsL150 relA1 ptsF rbs flbB5301*	[58]
DH5α	φ80d*lac*ZΔM15, *recA1*, *endA1*, *gyrA96, thi*-1, *hsdR*17(rK^-^,mK+) *supE44*, *relA1*, *deoR*, Δ(*lacZYA*-*argF*) U169	Promega
M15	F^-^, *lac*, *ara*, *gal*, *mtl*	[59]
JARV16-P	As MC4100 Δ*tatA*Δ*tatE pcn*B1 *zad*-981::Tn*10*d (Km^r^)	[8]
BØD-P	As MC4100 Δ*tatB pcn*B1 *zad*-981::Tn*10*d (Km^r^)	[8]
B1LK0-P	As MC4100 Δ*tatC pcn*B1 *zad*-981::Tn*10*d (Km^r^)	This work
DADE-P	As MC4100 Δ*tatABCD *Δ*tatE pcn*B1 *zad*-981::Tn*10*d (Km^r^)	[60]
Plasmids		
pREP4	Kan^R^, *lacI*^+^	Roche
pBluescript (IIKS^+^)	Amp^R^	Stratagene
pFAT415	pBluescript carrying *E. coli tatA*	[8]
pFAT416	pBluescript carrying *E. coli tatB*	[8]
pFAT417	pBluescript carrying *E. coli tatC*	[8]
pUniprom	Amp^R ^contains *E. coli tat *promoter and multiple cloning site	[51]
pUniprom-PA	pUniprom carrying *P. syringae tatA *gene	This work
pUniprom-PB	pUniprom carrying *P. syringae tatB *gene	This work
pUniprom-PC	pUniprom carrying *P. syringae tatC *gene	This work
pUniprom-PABC	pUniprom carrying *P. syringae tatABC *genes	This work
pUniprom-SA	pUniprom carrying *S. coelicolor tatA *gene	This work
pUniprom-SB	pUniprom carrying *S. coelicolor tatB *gene	This work
pUniprom-SC	pUniprom carrying *S. coelicolor tatC *gene	This work
pFATAQ1	pBluescript carrying *A. aeolicus tatA1*and *tatA2*	This work
pFATAQ2	pBluescript carrying *A. aeolicus tatBC*	This work
pFATAQ3	pBluescript carrying *A. aeolicus tatA*, *tatA2 *and *tatBC*	This work
pQE70	C-terminal His-tag expression vector	Qiagen
pQEAQ1	pQE70 carrying *A. aeolicus tatA1*	This work
pQEAQ2	pQE70 carrying *A. aeolicus tatA2*	This work
pQEAQBC	pQE70 carrying *A. aeolicus tatBC*	This work
pQEAQ12	pQE70 carrying *A. aeolicus tatA1*and *tatA2*	This work
